# IL-37 increases in patients after ischemic stroke and protects from inflammatory brain injury, motor impairment and lung infection in mice

**DOI:** 10.1038/s41598-019-43364-7

**Published:** 2019-05-06

**Authors:** Shenpeng R. Zhang, Marcel F. Nold, Sung-Chun Tang, Christine B. Bui, Claudia A. Nold, Thiruma V. Arumugam, Grant R. Drummond, Christopher G. Sobey, Hyun Ah Kim

**Affiliations:** 1 Department of Physiology, Anatomy & Microbiology, School of Life Sciences, La Trobe University, Bundoora Victoria, Australia; 2 Cardiovascular Disease Program, Biomedicine Discovery Institute and Department of Pharmacology, Monash University, Clayton Victoria, Australia; 3 Ritchie Centre, Hudson Institute of Medical Research, Melbourne Victoria, 3168 Australia; 4 Department of Paediatrics, Monash University, Melbourne Victoria, 3168 Australia; 5 Department of Neurology, National Taiwan University Hospital, Taipei, 10002 Taiwan; 6 Department of Physiology, Yong Loo Lin School of Medicine, National University of Singapore, Singapore, Singapore; 7 School of Pharmacy, Sungkyunkwan University, Seoul, South Korea

**Keywords:** Stroke, Neuroimmunology

## Abstract

Post-stroke inflammation may contribute to secondary brain injury and systemic immunosuppression. Interleukin(IL)-37 is an immunosuppressive cytokine belonging to the IL-1 superfamily with no mouse homologue yet identified, the effects of which have not been studied in stroke. Here we report: (1) the effect of ischemic stroke on circulating IL-37 in humans; and (2) the effect of IL-37 on stroke outcome measures in mice transgenic for human IL-37 (IL-37tg). We found that in the first 3 days after ischemic stroke in 55 patients, the plasma abundance of IL-37 was ~2-fold higher than in 24 controls. In IL-37tg mice, cerebral ischemia-reperfusion resulted in marked increases in plasma IL-37 (~9-fold) and brain *IL-37* mRNA (~7,000-fold) at 24 h compared with sham-operated IL-37tg mice. Further, compared with wild-type (WT) mice subjected to cerebral ischemia-reperfusion, IL-37tg mice exhibited less severe locomotor deficit, smaller cerebral infarcts and reduced bacterial lung infection. In the ischemic hemisphere, there were 60% fewer pro-inflammatory microglia-macrophages and up to 4-fold higher expression of anti-inflammatory markers in IL-37tg compared to WT mice. Our data show that IL-37 expression is increased following ischemic stroke in humans and IL-37tg mice, and may exert protective effects by modulating post-stroke inflammation in the brain and periphery.

## Introduction

Stroke is a leading cause of death and disability, with more than 800,000 cases occurring annually in the USA alone^1^. Ischemic stroke accounts for 87% of stroke cases, and is caused by the occlusion of a cerebral artery by a thrombus or embolus, resulting in neuronal death in the directly affected brain region^1^. Currently, the only available treatments are recombinant tissue plasminogen activator (rt-PA) within 4.5 h of stroke onset and/or mechanical clot retrieval within 8 h in the presence of either rt-PA or anticoagulants. Due to these time constraints, only ~15% of ischemic stroke patients can receive any such treatment^2–4^. In order to identify novel therapeutic targets for development of additional stroke therapies with wider application, we will need to first gain a better understanding of mechanisms and mediators that may influence stroke outcome.

Post-stroke inflammation has been well documented in animal models, and is believed to contribute to secondary brain injury^5,6^. Inflammation is initiated by the activation of resident microglia, followed by increases in cell adhesion molecules and chemokines, leading to the infiltration of immune cells through the blood-brain barrier^5,6^. Both innate and adaptive immune responses contribute to the post-ischemic inflammation. Several types of inflammatory cells enter the brain parenchyma and may release pro-inflammatory mediators, such as cytokines and reactive oxygen species, and thus promote further tissue damage and infarct enlargement^7–9^. Some infiltrating immune cells, such as T regulatory cells and M2-polarized microglia-macrophages, can instead exhibit anti-inflammatory characteristics in the post-stroke brain^9–11^.

Inflammation after stroke may also be associated with altered systemic immune function, lymphopenia and splenic atrophy^12,13^, and can render individuals susceptible to infection^14,15^. Indeed, ~30% of fatalities in stroke patients are associated with infections, especially in the form of pneumonia^16,17^. Thus, an understanding of the mechanisms of post-ischemic inflammation is crucial to identify potential therapies.

Here, we have investigated a relatively newly identified cytokine named IL-37 (previously known as IL-1F7)^18,19^ belonging to the IL-1 superfamily, and its regulation and action in stroke. IL-37 is currently the only IL-1 family cytokine of which a murine homologue has not yet been identified^19,20^. In humans and also mice, IL-37 binds to IL-18Rα, forming a complex with IL-1R8 (SIGIRR) and may act either intracellularly or extracellularly on a variety of tissues including brain where the IL-37a isoform is thought to be preferentially expressed^21–23^. While IL-37b – the isoform expressed by these mice – is the most studied isoform, a total of five IL-37 isoforms (a–e) exist and are distributed differentially in various organs and tissues in humans^18^. Unlike most other IL-1 family members, IL-37 is anti-inflammatory and its mRNA is inducible in several innate and adaptive immune cell types such as monocytes, neutrophils, natural killer cells, as well as plasma cells^18,19,22,24,25^. Effects of IL-37 have been investigated in endotoxic shock^19^, inflammatory bowel disease^26^, myocardial infarction^27^, spinal cord injury^28^, multiple sclerosis^29^ and liver ischemia^30^. While a recent pilot study reported IL-37 to be elevated in serum of 5 ischemic stroke patients^31^, there is no information on regulation or effect of IL-37 in the setting of stroke. Thus, we have investigated: (1) the abundance of IL-37 in the blood of control and ischemic stroke patients, and in post-mortem brain sections; and (2) the effect of IL-37 on stroke outcome measures in mice transgenic for human IL-37 (IL-37tg). The mRNA encoding *IL-37* contains an instability sequence such that, even under the control of the constitutively active CMV promoter in these IL-37tg mice, an inflammatory stimulus (e.g. as in the ischemic brain) is required for mRNA upregulation and thus protein production^19,26,28,32^.

## Results

### IL-37 expression is elevated in brain and plasma after acute ischemic stroke

**Figure 1:**
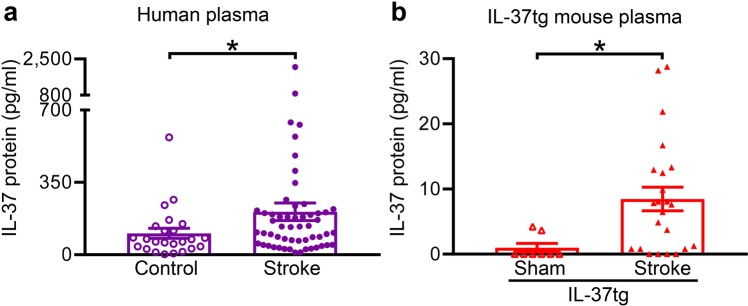
Ischemic stroke increases IL-37 in the plasma level of humans and mice. (**a**) Circulating level IL-37 protein is shown for control subjects (n = 24) and patients 3 days after an ischemic stroke (n = 55). (**b**) IL-37 protein abundance is shown for IL-37tg mice at 24 h after either sham surgery (n = 8) or ischemic stroke (n = 23). Data are presented as mean ± S.E.M.; *P < 0.05, Welch’s t-test.

To investigate the regulation of IL-37 in the setting of stroke, we explored IL-37 protein and mRNA in humans after stroke and in mice in a model of this disease. In patients with acute ischemic stroke, the plasma abundance of IL-37 was approximately double that of control patients 3 days after the event (*P* = 0.038; Fig. 1a), but there was no correlation with two indicators of stroke severity – the National Institute of Health Stroke Scale (NIHSS, ρ = −0.14, *P* = 0.304) or Glasgow Coma Scale scores (ρ = 0.10, *P* = 0.488). Post-stroke plasma IL-37 abundance on day 3 was similar to that recorded within 2 days of stroke onset (170 ± 245 pg/ml). Similarly, in IL-37tg mice subjected to ischemic stroke, plasma IL-37 was ~8-fold higher than in sham-operated IL-37tg mice 24 h after surgery (*P* < 0.001; Fig. 1b; note that WT mice do not express IL-37). As in human patients after stroke, plasma IL-37 was not correlated with post-stroke clinical scores in mice (ρ = 0.07, *P* = 0.733).

**Figure 2:**
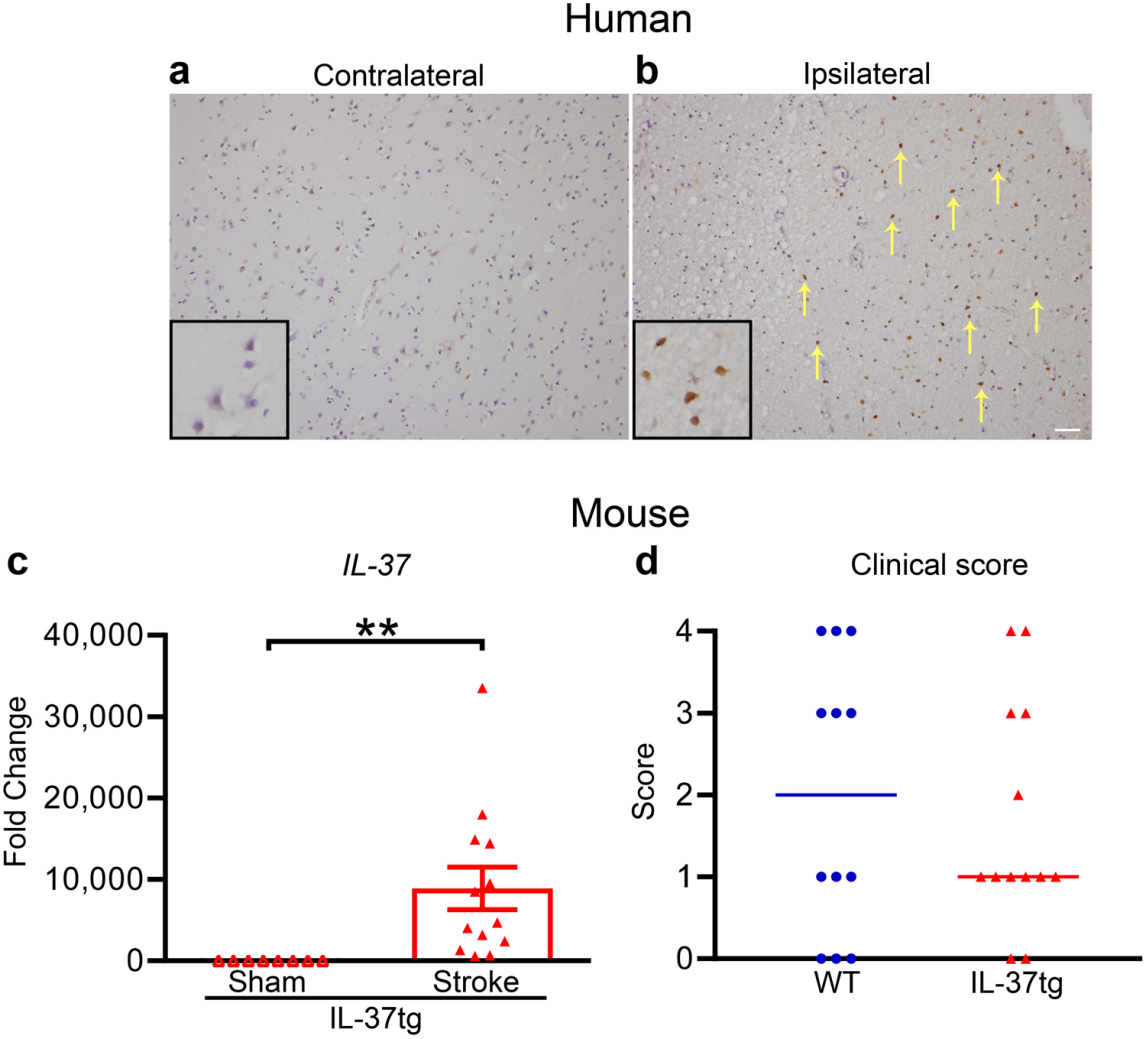
IL-37 is increased in the brain after stroke in a human and in IL-37tg mice, and is associated with a lower median clinical score in mice. Photomicrographs showing IL-37 immunoreactivity is (**a**) absent in a brain section from the contralateral hemisphere and (**b**) present (arrows) in the ischemic hemisphere of a stroke patient. Scale bar = 100 µm. (**c**) Expression of *IL-37* mRNA in brains of IL-37tg mice 24 h after sham surgery (n = 8) or in the ischemic hemisphere after stroke (n = 13). The mean of the sham group is set as 1.0. **P < 0.01; Welch’s t-test. (**d**) Clinical score for wild-type (WT; n = 12) and IL-37tg mice (n = 13) at 24 h after stroke (*P* = 0.747; Mann-Whitney test). Data in (**c**,**d**) are presented as mean ± S.E.M. or median, respectively.

Immunohistochemical analysis of post-mortem human brain sections obtained from a person who died after ischemic stroke indicated IL-37-expressing cells accumulating within the infarct but not in the non-ischemic hemisphere (Fig. 2a,b). We were unable to perform immunohistochemical analysis of IL-37 in mouse brain due to cross-reactivity of the antibody in WT tissue (not shown). However, brain expression of *IL-37* mRNA was profoundly increased in IL-37tg mice specifically in the ischemic hemisphere after stroke (~7,000-fold; *P* = 0.005; Fig. 2c). These IL-37tg mice also had a lower median clinical score than concurrently studied WT counterparts subjected to stroke on the same day (Fig. 2d). Furthermore, there was a strong trend towards a negative correlation between brain expression of *IL-37* mRNA and clinical score in mice after stroke (ρ = −0.50, *P* = 0.085), whereas this was not the case for plasma IL-37 protein (ρ = −0.07, *P* = 0.833).

### Effect of IL-37 on stroke outcomes

Following insertion of the monofilament to occlude the middle cerebral artery (MCA) and induce cerebral ischemia, blood flow to the region of cortex supplied by the MCA was reduced by ~75% in both WT and IL-37tg mice. Rapid, near-complete reperfusion was achieved in all mice following monofilament withdrawal, and the presence of IL-37 had no effect on changes in regional cerebral blood flow (rCBF) (Supplementary Fig. S1). No anatomical differences were observed between WT and IL-37tg mice upon examination of gross anatomy of the cerebral vasculature, including the Circle of Willis and its branches (not shown). We observed no difference in post-stroke mortality at 24 h, with deaths in 16.3% (7/43) of WT and 15.9% (7/44) of IL-37tg mice.

**Figure 3:**
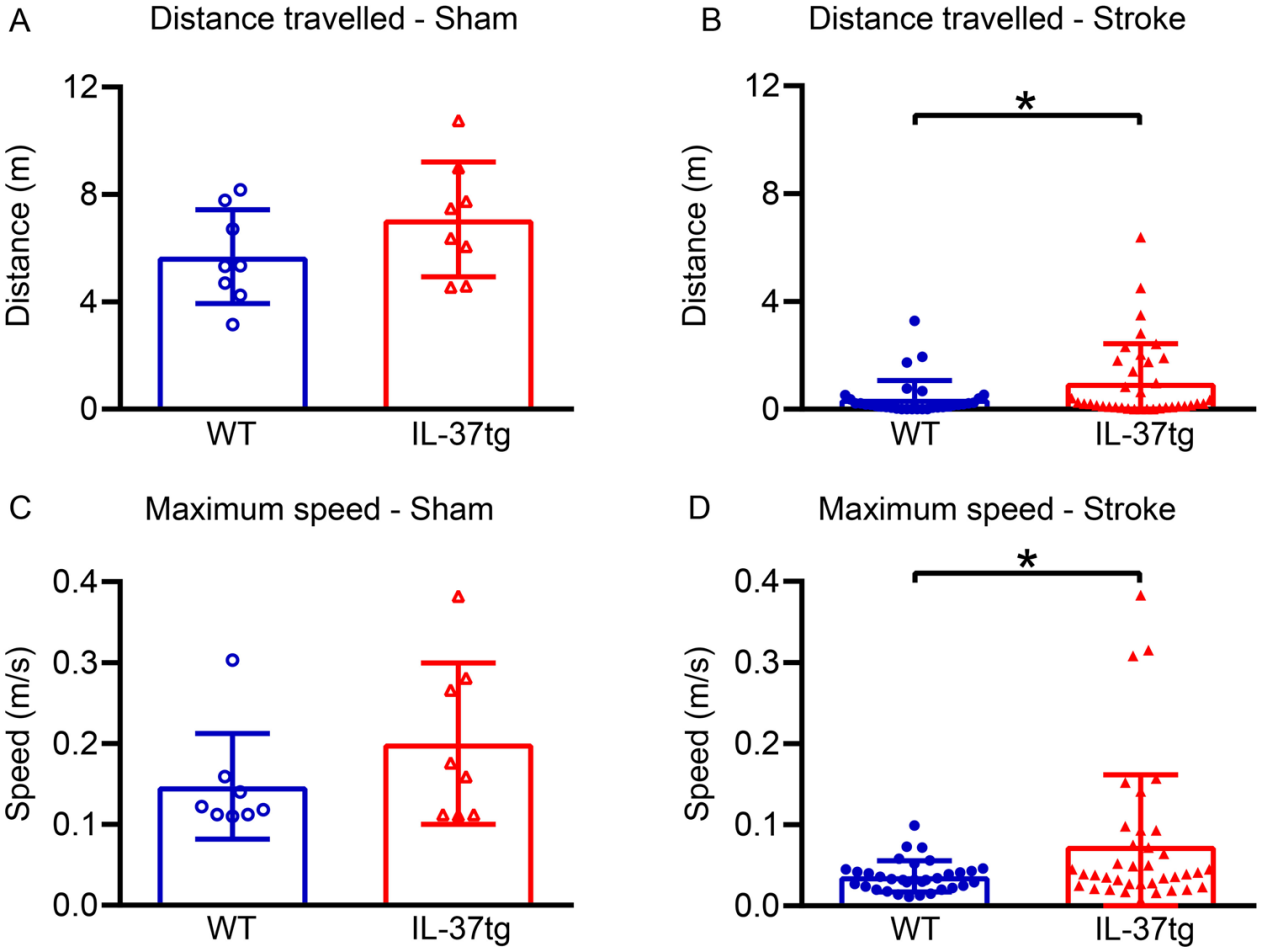
Post-stroke functional deficit is less in IL-37tg mice. Measures of post-stroke mobility, namely total distance travelled in 5 min (**A**,**B**) and maximum speed attained (**C**,**D**), were obtained 24 h after sham surgery (**A**,**C**; n = 8 for each group) or after stroke surgery (**B**,**D**; n = 37 for IL-37tg and n = 33 for WT). Data are shown as mean ± S.E.M; *P < 0.05, unpaired Student’s t-test.

**Figure 4:**
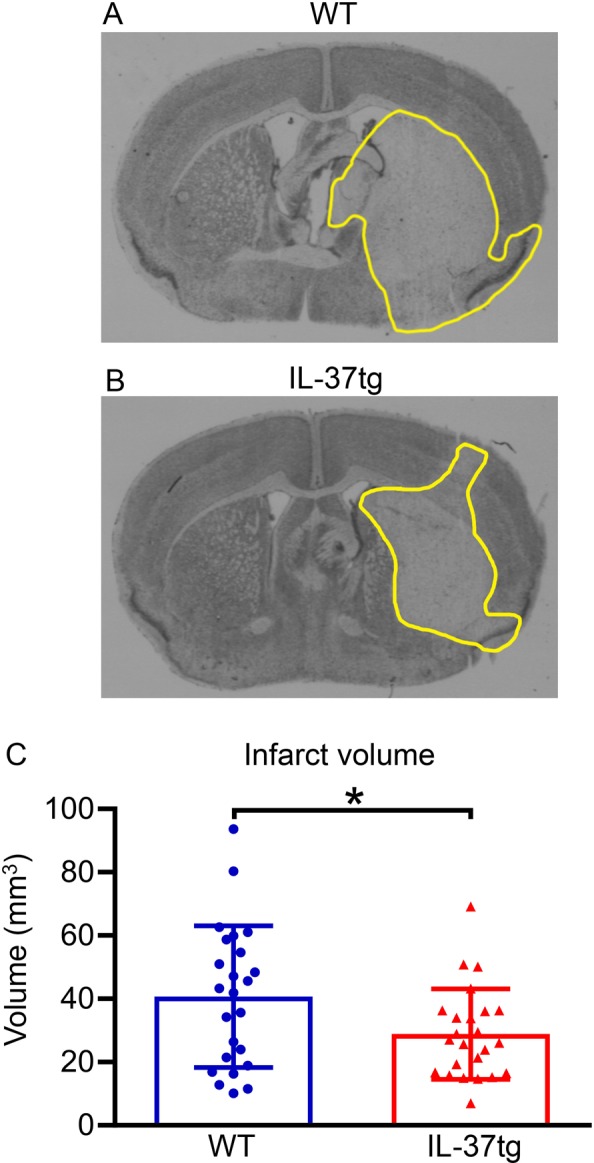
Infarct volume is reduced in IL-37tg mice after stroke. Coronal brain sections are shown from (**A**) a WT and (**B**) an IL-37tg mouse 24 h post-stroke with the infarct perimeter delineated in yellow. Images are representative of n = 24 mice per group. (**C**) Total infarct volume is shown as mean ± S.E.M; *P < 0.05, Welch’s t-test.

Open field testing indicated no difference in motor function between WT and IL-37tg mice at 24 h after sham surgery (*P* = 0.176 and *P* = 0.230; Fig. 3A,C, respectively). By contrast, virtually all mice subjected to stroke exhibited deficits in motor function (Fig. 3B,D). We observed that these functional deficits were significantly less severe in IL-37tg than in WT mice following stroke; for example, IL-37tg mice travelled 2.5 times further (*P* = 0.036) and achieved a 2-fold higher maximum speed (*P* = 0.019) than WT mice after stroke (Fig. 3B,D). Cerebral infarct volume was ~30% smaller in IL-37tg versus WT mice (*P* = 0.034; Fig. 4A–C), particularly in the subcortex (Supplementary Fig. S1).

### Regulation of anti-inflammatory mediators in mouse brain after stroke

**Figure 5:**
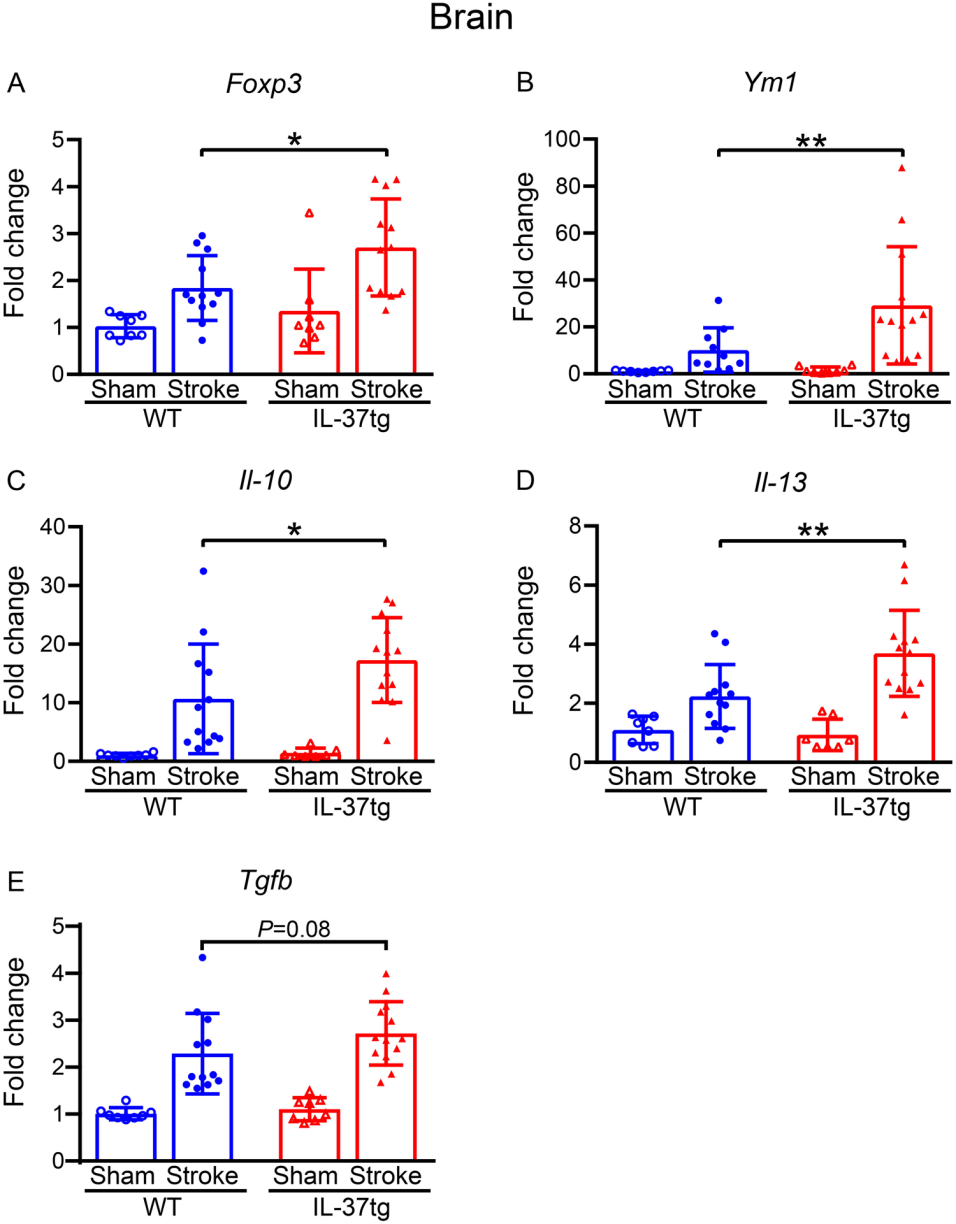
IL-37 increases anti-inflammatory cytokines in the brain after stroke. (**A**) *Foxp3*, (**B**) *Ym1*, (**C**) *Il-10*, (**D**) *Il-13* and (**E**) *Tgfb* were quantified by qPCR in the ischemic hemisphere of WT and IL-37 tg mice (n = 8–13) 24 h after stroke. Data are presented as mean ± S.E.M.; *P < 0.05 and **P < 0.01, one-way ANOVA with Bonferroni post-hoc test.

We examined mRNA levels of several inflammation-related genes known to be associated with stroke in the brain. As seen for *IL-37* (Fig. 2a), several other anti-inflammatory markers, namely *Foxp3 (P* = 0.012), *Ym1 (P* = 0.006), *Il-10 (P* = 0.018*)*, *Il-13 (P* = 0.002) and *Tgfb (P* = 0.008) were up to 3-fold increased in the ischemic hemisphere following stroke in IL-37tg compared to WT mice (Fig. 5A–E).

### Effect of IL-37 on brain infiltration of immune cells following stroke

**Figure 6:**
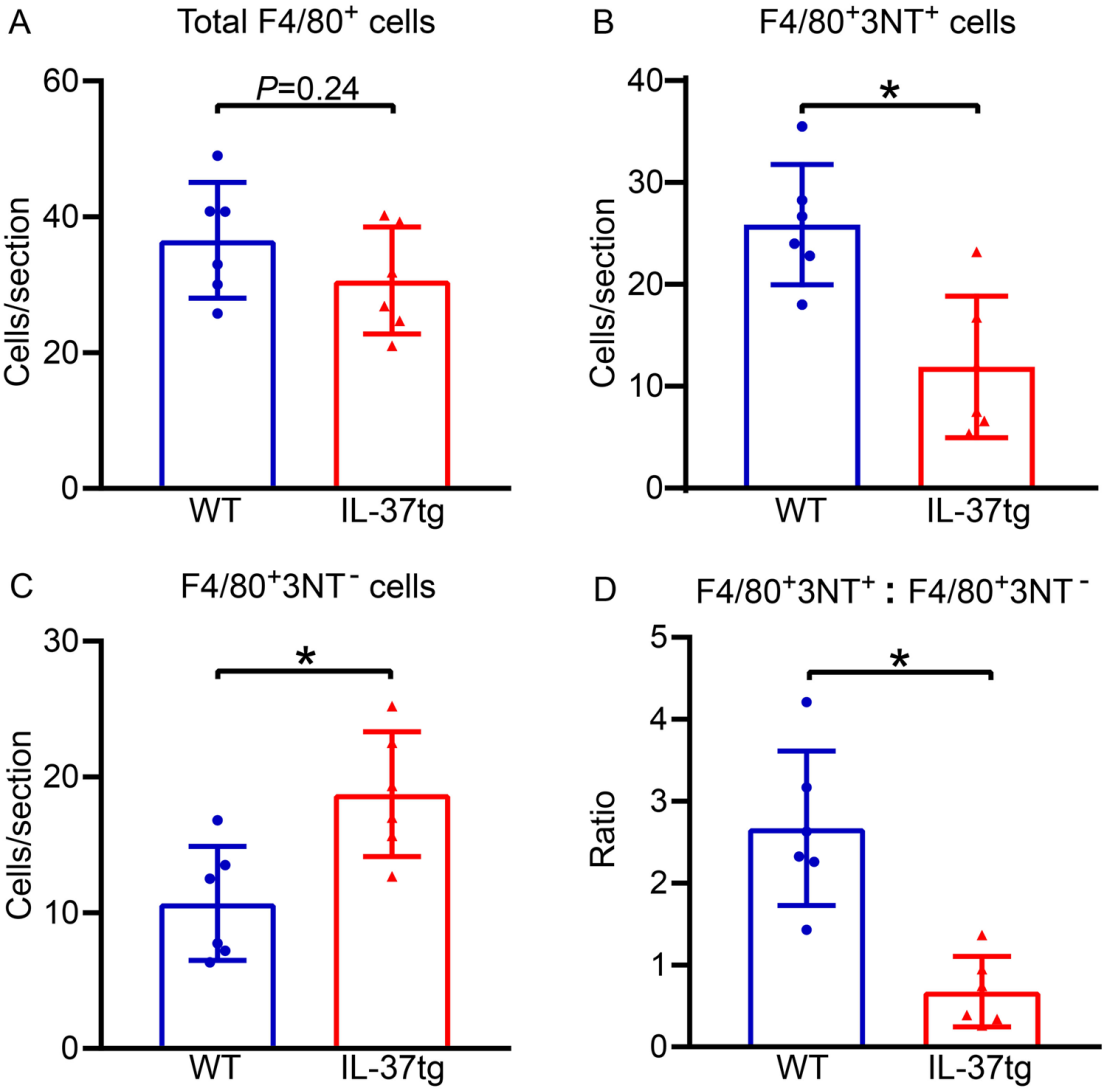
IL-37 reduces pro-inflammatory, and increases anti-inflammatory microglia-macrophages in the brain after stroke. (**A**) Immunohistochemical quantification of total microglia-macrophages (F4/80^+^) in the ischemic hemisphere 24 h after stroke. Also shown are numbers of (**B**) pro-inflammatory (3-nitrotyrosine[3-NT]-positive) and (**C**) anti-inflammatory (3-NT-negative) F4/80^+^ cells, and (**D**) the ratio of F4/80^+^3-NT^+^ to F4/80^+^3-NT^−^ microglia-macrophages in WT and IL-37tg mice. All data are n = 6 and are presented as mean ± S.E.M.; *P < 0.05, unpaired Student’s t-test.

Immunohistochemistry revealed that there were similar numbers of CD45^+^ leukocytes, neutrophils (MPO^+^), T cells (CD3^+^), astrocytes (GFAP^+^) (Supplementary Fig. S2) and microglia-macrophages (Fig. 6A) in the ischemic hemisphere of WT compared with IL-37tg mice after stroke. However, using immunohistochemistry we observed that the activation/polarization states of microglia-macrophages in the ischemic hemisphere were markedly different between IL-37tg and WT mice: IL-37tg mice exhibited 55% fewer pro-inflammatory cells (F4/80^+^3-NT^+^; *P* = 0.004; Fig. 6B) and 73% more alternatively activated cells (F4/80^+^3-NT^−^; *P* = 0.010; Fig. 6C) than WT mice, which reflected a ratio strongly polarized towards an ‘alternative activation’ pattern (*P* < 0.001; Fig. 6D).

### Effect of IL-37 on post-stroke lung infection and anti-inflammatory cytokines

**Figure 7:**
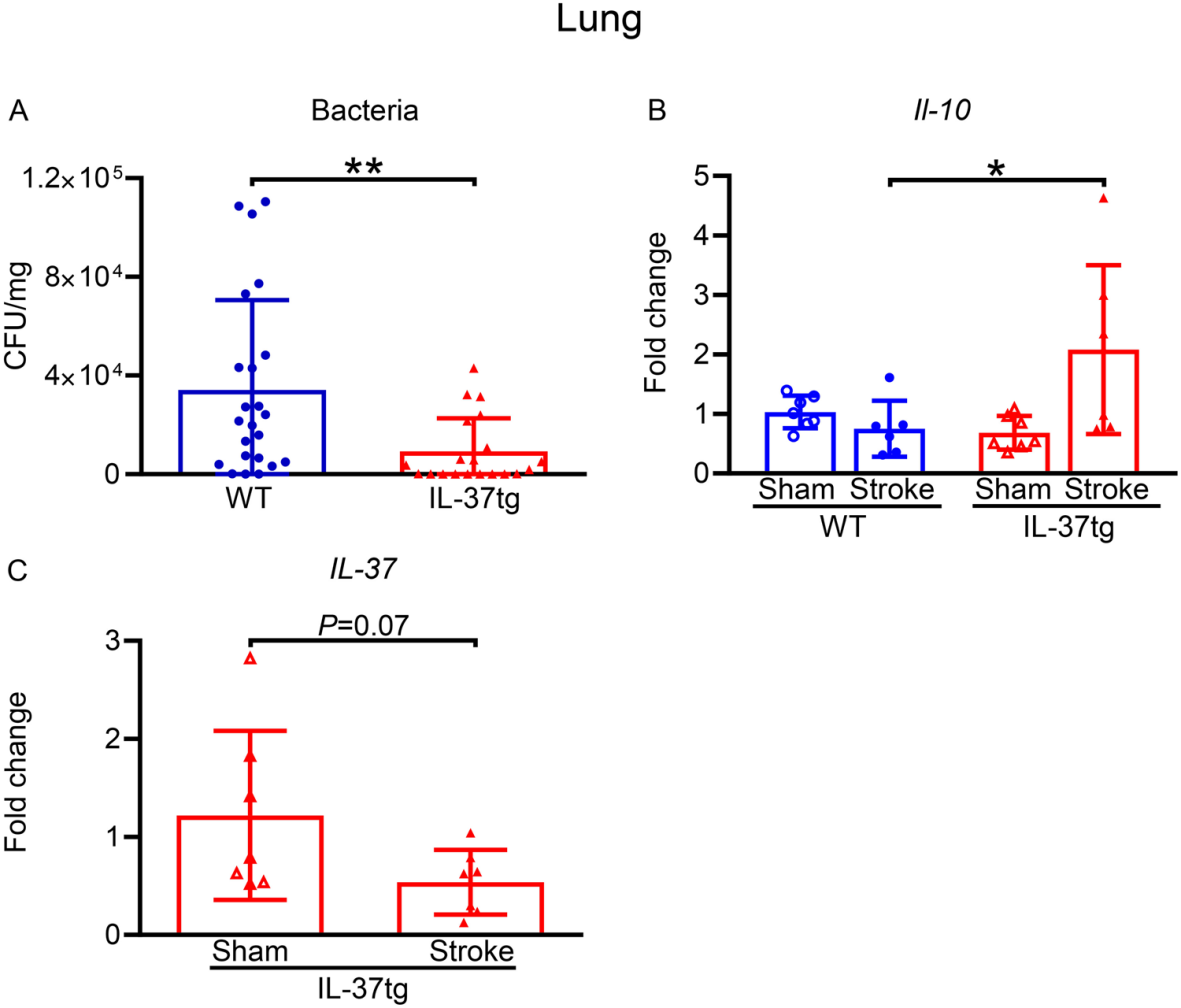
Post-stroke lung infection is reduced in IL-37tg mice. (**A**) Bacterial load in the lung of WT (n = 23) and IL-37tg (n = 20) mice, and expression of (**B**) *IL-37* and (**C**) *Il-10* (n = 6–7) mRNA 24 h after stroke are shown. CFU, colony forming unit. Data are presented mean ± S.E.M.; *P < 0.05 and **P < 0.01, unpaired Student’s t-test.

Targeting post-stroke brain inflammation with anti-inflammatory therapy may be neuroprotective, but may entail detrimental systemic consequences, such as increased lung infection^15^. However, lungs of IL-37tg mice exhibited 73% fewer bacterial colony forming units than WT mice after stroke (*P* = 0.005; Fig. 7A). This was associated with 2.7-fold greater lung expression of *Il-10* mRNA (*P* = 0.006; Fig. 7B), whereas in contrast to brain (i.e. Fig. 2c), *IL-37* mRNA in the lungs tended to be reduced by stroke (*P* = 0.075; Fig. 7C).

## Discussion

There are five major findings of this study. First, ischemic stroke resulted in a marked increase in plasma IL-37 in both humans and IL-37tg mice. Second, this was associated with augmented abundance of IL-37 in the ischemic brain tissue of one stroke patient and in mice. Third, compared with WT mice subjected to cerebral ischemia-reperfusion, IL-37tg mice exhibited less severe locomotor deficit and smaller cerebral infarcts. Fourth, the neuroprotection evident in IL-37tg mice involved fewer pro-inflammatory microglia-macrophages and higher expression of anti-inflammatory cytokines in the ischemic brain hemisphere. Fifth, post-stroke bacterial lung infection was profoundly reduced in IL-37tg compared to WT mice.

It is known that activation of certain anti-inflammatory signalling pathways can promote neuroprotection following stroke, but may not necessarily avoid adverse systemic effects^17^. For example, previously we found that systemic administration of IL-33, a Th2-promoting cytokine, is neuroprotective, but these beneficial effects were offset by accelerated systemic immunosuppression and augmented bacterial infections^15^. In contrast to IL-33, IL-37 is inducible by a broad range of pro-inflammatory mediators, including cytokines such as those generated in the brain following ischemic stroke^33–36^. As a powerful suppressor of innate and adaptive immunity, it then reduces non-specific, T helper (Th) 1, Th2 and Th17 immune responses via negative feedback inhibition^19–21,37–40^. Here we assessed the effect of ischemic stroke in humans on the regulation of IL-37 in blood and the post-mortem brain. Furthermore, we utilised transgenic mice for human IL-37 to similarly assess the effect of stroke on IL-37 regulation in that species, and to investigate the effect of IL-37 on brain injury, motor impairment and bacterial infection following stroke. Unlike humans, these IL-37tg mice express IL-37b driven by the CMV promotor. Expression of this splice variant is therefore ubiquitous in these mice, including in the brain cells or in immune cells infiltrating the brain, thus allowing us to investigate the effects of IL-37 in various disease settings including stroke.

Our data show that plasma IL-37 was increased following stroke in both humans and IL-37tg mice, and that brain (but not lung) abundance of IL-37 was also augmented, consistent with its production being driven by pro-inflammatory signalling^19,41,42^. We noted that a lower clinical score in mice was more strongly related to expression of *IL-37* mRNA in the brain than with circulating IL-37 protein, consistent with the concept that IL-37 generated locally in the brain is likely to be the cause of neurological protection. Likely cellular sources of plasma IL-37 are peripheral blood mononuclear cells, including monocytes-macrophages and dendritic cells^43^, and brain expression of IL-37 may occur in astrocytes, microglia and infiltrating macrophages^28,35^. We were unable to perform IL-37 co-localisation study with these immune cells due to the cross-reactivity of human IL-37 antibody on mouse tissues. Indeed, it was noteworthy that whereas transgenic expression of IL-37 had no effect on total numbers of immune cells infiltrating the post-ischemic mouse brain, there was a marked reversal in the ratio of pro-inflammatory (3-NT^+^; indicative of peroxynitrite-induced oxidative damage in pro-inflammatory microglia-macrophages) to anti-inflammatory (3-NT^−^) microglia-macrophages (F4/80^+^ cells)^10,13^ in IL-37tg compared with WT mice. These findings, which were associated with higher expression of anti-inflammatory markers in the ischemic brain, are consistent with reports that IL-37 reduces the pro-inflammatory effects of immune cells, especially macrophages^19,44,45^.

MCA occlusion-reperfusion in WT and IL-37tg mice produced a similar rCBF profile and thus equivalent severity of ischemia, but it resulted in a milder degree of functional impairment and a smaller infarct volume in IL-37tg mice. These protective effects of IL-37 are analogous to the greater residual locomotor function displayed by IL-37tg mice after spinal cord injury^28^. Here, the amelioration of locomotor deficit and brain injury were associated with less bacterial infection of the lungs after stroke at 24 h following stroke, a timepoint at which we find post-stroke lung infection in mice to be maximal. Previous studies^15,46^ have shown that post-ischemic functional outcome (i.e. clinical score and locomotor activity) after stroke does not strictly correlate with cerebral infarct volume; rather, other factors such as lung infection can contribute significantly to post-stroke morbidity^14^. It is therefore likely that the multifactorial benefits of IL-37, including neuroprotection and reduction of lung infection, produced the improvement of functional outcome in combination. It has been suggested that the post-stroke lung infections are the result of bacteria translocating from a leaky intestinal epithelium^47,48^. Since IL-37 has been demonstrated to ameliorate intestinal inflammation and protect barrier functions^26,40^, it is tempting to speculate that such protection of the intestine may contribute to the rescue of IL-37tg mice from bacterial infection in the lungs.

There are some limitations of this study. Firstly, WT and IL-37tg mice were housed separately as controls. Future studies that include co-housing prior to study to synchronise microbiomes could assess the role of the gut microbiome environment in our findings. Secondly, here we investigated the effect of IL-37 on post-stroke outcome at 24 h only, and it will be important to clarify if the IL-37-dependent protective effects are sustained over a longer period. Thirdly, as this study was restricted to young to middle aged adult male mice, future studies should evaluate the effects of IL-37 in females and also in aged mice of both sexes.

In summary, this study has provided the first data assessing the effect of ischemic stroke on the regulation of IL-37 in humans and IL-37tg mice, and of the impact of IL-37 on stroke outcome measures. Our findings reveal that IL-37 is augmented in the brain and plasma following ischemic stroke, and exerts protection by modulating post-stroke inflammation in the brain and periphery. IL-37 may therefore have potential as a novel therapeutic approach in stroke, and future work to explore the therapeutic potential of post-stroke administration of recombinant IL-37 is therefore warranted.

## Material and Methods

### Patients

The study was approved by the National Taiwan University Hospital Committee of Human Research and conducted in accordance with the Helsinki Declaration of 1975 (and as revised in 1983). Written informed consent was obtained from the patients or from the next of kin of patients with decreased consciousness. The study included 55 ischemic stroke patients admitted within 24 h of symptom onset. Head MRI or repeated CT examination was performed at admission and at 24 h after symptom onset to confirm the diagnosis of acute ischemic stroke. Patients were excluded if they had received rt-PA or had active infection, autoimmune disease or were under steroid therapy. The study also included 24 control subjects matched for sex and age who were free of cerebrovascular disease for >12 months. Venous blood was obtained from controls or from patients on day 3 after stroke onset. Clinical information including stroke presentation, risk factors, co-morbidities, blood pressure, plasma lipid data (see below) were collected at the time of admission from all study subjects. The severity of stroke was assessed by the National Institutes of Health Stroke Scale (NIHSS) and the Glasgow Coma Score (GCS). Patient characteristics are shown in Table 1.

**Table 1:** Patient characteristics.

Patient Characteristics	Control (N = 24)	Acute stroke (N = 55)	*P* value
Age (years; mean ± SD)	72 ± 8	68 ± 14	0.247
Males – number (%)	19 (79)	36 (65)	0.292
NIHSS at admission no./total no. (%)	—		
0	—	0/55 (0)	—
1–4	—	10/55 (18)	—
5–15	—	19/55 (35)	—
16–20	—	10/55 (18)	—
21–42	—	16/55 (29)	—
Glasgow Coma Scale at admission no./total no. (%)	—		
1–8	—	5/50 (10)	—
9–12	—	18/50 (36)	—
13–15	—	27/50 (54)	—
Systolic blood pressure SBP (mmHg; mean ± SD) at admission	—	154 ± 29	—
Diastolic blood pressure DBP (mmHg; mean ± SD) at admission	—	91 ± 21	—
Blood pressure classification* no./total no. (%)			
Normal	—	4/52 (8)	—
Elevated	—	4/52 (8)	—
Hypertension stage 1	—	6/52 (11)	—
Hypertension stage 2	—	38/52 (73)	—
Smoking – no./total no. (%)	4/14 (29)	23/54 (43)	0.379
Diabetes mellitus no./total no. (%)	8/24 (33)	25/54 (37)	0.748
High plasma triglyceride (≥150 mg/dl) no./total no. (%)	11/24 (46)	18/54 (33)	0.803
High plasma total cholesterol (≥200 mg/dl) no./total no. (%)	6/24 (25)	18/54 (33)	0.239

*Normal: SBP < 120 mmHg and DBP < 80 mmHg; Elevated: SBP 120–129 mmHg and DBP < 80 mmHg; Hypertension stage 1: SBP 130–139 mmHg or DBP 80–89 mmHg; Hypertension stage 2: SBP > 140 mmHg or DBP > 90 mmHg.

### Human blood and brain tissue

Venous blood samples (n = 79) were collected from control patients (n = 24) or ischemic stroke patients on day 3 after stroke onset (n = 55). Controls included here were stable patients of the clinic who had an ischemic stroke >2 years previously. Blood was immediately placed into EDTA-treated anticoagulation tubes and centrifuged at 300 × *g* for 15 min at 4 °C to obtain plasma, which was removed with a Pasteur pipette and immediately frozen at −80 °C for future analysis. The plasma levels of IL-37 were determined using a commercially available ELISA kit (see below).

A sample of human brain tissue was obtained from an anonymized autopsy patient in National Taiwan University Hospital with approval from the National Taiwan University Hospital ethics committee. The patient was a 38 year-old female who had acute myocarditis. Right hemispheric infarct developed during the admission with mortality outcome. The brain tissue was collected within 48 h of death and specimens were fixed in 4% buffered formalin for at least 3 weeks before paraffin embedding, then sections were stained according to standard immunohistochemistry procedures described below using primary antibodies against IL-37 (Clone: 37D12; Thermal Fisher Scientific, USA) and the Dako REAL™ EnVision™ Detection System (Dako, Denmark). Briefly, brain sections were deparaffinized by heating at 60 °C for 30 min followed by xylene application. Sections were then rehydrated by passing through a series of decreasing concentrations of ethanol (100%, 90%, 70% and 50%) for 5 min each step. Sections were then washed in 0.1 M of PBS. Endogenous peroxidase was quenched with 3% hydrogen peroxidase for 10 min. Sections were then blocked with IL-37 antibody (1:100) at 4 °C overnight. Sections were washed the following day, and stained with Dako REAL™ EnVision™/HRP, Rabbit/Mouse (ENV). Dako REAL™ DAB+ Chromogen was applied to sections after washing. Sections were dehydrated with ethanol and xylene prior to being mounted with DPX mountant (VWR International, USA).

### Animals

A total of 147 male mice (average age = 17.6 weeks) were used in this study. Homozygous mice transgenic for human IL-37 (IL-37tg) were generated as described previously^19^. Our IL-37tg mouse colony was bred from the original colony, which was backcrossed onto C57BL/6 mice for >10 generations. To generate the mice, fertilised eggs from C57BL/6 mice were injected with a pIRES IL-37b expressing plasmid and genotypes were identified at the age of 3–4 weeks^19^. Animals studies have been reported in full compliance with the ARRIVE guidelines^49^. Both C57Bl/6 (WT: n = 71) and IL-37tg mice (n = 76) were bred at Monash Animal Research Platform, and were housed in separated neighbouring boxes in Monash Animal Research Laboratory prior to experimentation. Of these, 38 were excluded from the study because of: (1) death during the surgical procedure (WT, n = 9; IL-37tg, n = 3); (2) <50% reduction in rCBF relative to the pre-occlusion level during cerebral artery occlusion (WT, n = 7; IL-37tg, n = 15); or (3) subarachnoid hemorrhage due to filament insertion (WT, n = 1; IL-37tg, n = 3). Mice were housed in a specific pathogen-free environment with free access to food and water. All experiments were approved by Monash University Animal Ethics Committee (Project MARP/2014/064 and MARP/2016/038) and performed in accordance with the Australian Code for the Care and Use of Animals for Scientific Purposes, National Health and Medical Research Council of Australia. Animal characteristics are shown in Table 2.

**Table 2:** Animal characteristics.

Animal Characteristics	WT (N = 54)	IL-37tg (N = 53)	*P* value
Age (weeks)	17.9 ± 0.7	17.6 ± 0.8	0.559
Pre-surgical weight (g)	33.6 ± 0.5	31.5 ± 0.6	0.012
Post-surgical weight (g)	31.5 ± 0.6	28.9 ± 0.5	0.003
Reduction in rCBF during MCAO (%)	73.5 ± 1.6	74.3 ± 1.5	0.723
Level of reperfusion relative to baseline (%)	96.7 ± 4.4	88.8 ± 5.2	0.256

rCBF, relative cerebral blood flow; MCAO, middle cerebral artery occlusion. Data are mean ± SD.

### Transient focal cerebral ischemia in mice

Mice were anesthetized with ketamine and xylazine (80 mg/kg and 10 mg/kg, respectively, i.p.). Rectal temperature was maintained at 37 ± 0.5 °C by a heat lamp throughout the surgery until the animal regained consciousness. Both sham and stroke surgical procedures have been described previously^15,46^. Ischemia was achieved by inserting a 6-0 silicone-coated nylon monofilament (Doccol, USA) into the distal internal carotid artery and advancing it to occlude the origin of the MCA. Reperfusion was achieved by retraction of the monofilament after 1 h of occlusion. Successful ischemia-reperfusion was confirmed with transcranial laser-Doppler flowmetry (Perimed, Sweden) in each animal, and was defined as >50% reduction of rCBF during ischemia and >50% of recovery of rCBF towards baseline within 10 min of reperfusion. rCBF was recorded for a further 30 min after reperfusion. Sham-operated mice were anesthetized and the right carotid bifurcation was exposed without insertion of a filament. Wounds were then closed, and mice were allowed to recover in a clean cage placed on a heating pad for 23 h. All mice were injected s.c. with 1 ml of sterile saline during their recovery.

### Assessment of motor activity and clinical score in mice

At 24 h after sham or MCA occlusion surgery, mice were subjected for 5 min to a modified open field with parallel rod floor test for which they were placed in an acrylic box (20 × 20 cm) with raised parallel metal rods across the floor, and locomotor data were automatically collected and analysed by the device software (ANY-maze, Stoelting, USA). Some mice (N = 12 for WT, N = 13 for IL-37tg) were assessed according to a five-point ‘clinical scoring’ system^50,51^, 0: normal motor function; 1: flexion of torso and contralateral forelimb when lifted by tail; 2: circling to one side when held by tail on a flat surface, but normal posture at rest; 3: leaning to one side at rest; 4: absence of spontaneous activity. Clinical scoring was performed by an investigator blinded as to the genotype and surgical protocol.

### Cerebral infarct and edema volume in mice

Following motor assessment, mice were killed by isoflurane inhalation overdose and decapitation. Brains were removed immediately and snap frozen in liquid nitrogen. Coronal brain sections (30 µm) separated by ∼420 µm were cut and thaw-mounted onto glass slides coated with poly-L-lysine. Sections were then stained with thionin (0.1%) to delineate the infarct area. Total, cortical, subcortical infarct and edema volumes were estimated as described previously^15,46^.

### Immunohistochemistry

Immunohistochemical analysis was performed in brain sections for several cell types, including pan leukocytes (CD45^+^), microglia-macrophages (F4/80^+^ that were positive or negative for 3-nitrotyrosine; 3-NT), T lymphocytes (CD3^+^), neutrophils (myeloperoxidase, MPO^+^) and astrocytes (GFAP^+^). Frozen coronal brain sections (10 µm) were thaw mounted onto poly-L-lysine coated glass slides. A 3,3′-diaminobenzidine (DAB) kit (Dako, Denmark) was used to detect CD3^+^ T cells or MPO^+^ neutrophils. Sections were air dried before fixing in ice-cold 4% paraformaldehyde for 15 min. Sections were then blocked with peroxidase for 10 min followed by washing in 0.01 M phosphate-buffered saline (PBS). Sections were then blocked in 10% normal goat serum for 30 min and incubated with primary antibodies overnight at room temperature (rabbit-anti-CD3, 1:200; Catalogue: ab215212,, or rabbit-anti-MPO, 1:100; Catalogue: ab188211, Abcam, UK). They were washed the next day, followed by staining with peroxidase-labelled polymer conjugated to goat anti-rabbit immunoglobulins for 2 h. DAB was applied onto sections after washing. All sections were then counterstained with 25% hematoxylin for 2 min and dehydrated with ethanol and xylene prior to being mounted with DPX mountant (VWR International, USA). Immunofluorescent staining was also performed on frozen brain sections to detect CD45^+^ leukocytes (Alexa Fluor 488 rat-anti-mouse CD45; Clone: 30-F11, Biolegend, USA) or GFAP^+^ astrocytes (rabbit anti-GFAP, 1:500; Catalogue: ab7260, Abcam, UK). For both, sections were stained with primary antibody (1:50 or 1:500, respectively) overnight at 4 °C. Following a wash the next day, GFAP-stained sections were incubated with secondary antibody (Alexa Fluor 594 goat anti-rabbit, 1:200; Catalogue: A-11034, Invitrogen, USA) for 2 h at room temperature prior to counterstaining with DAPI. Double immunohistochemical staining with 3-NT (1:50 Catalogue: ab61392, Abcam, UK) and F4/80 (1:100; Clone: Cl:A3-1, Bio-Rad, USA) was also performed to detect pro-inflammatory (i.e. ‘M1-polarized’) macrophages, as described previously^10,13,15^. Sections were washed in 0.01 M PBS, coverslipped, and examined using an Olympus fluorescence microscope. Numbers of CD3^+^, MPO^+^, F4/80^+^ or GFAP^+^ cells in the left (contralateral) and right (ischemic) hemispheres were counted in 6 brain sections per mouse by a researcher blinded to group identity. CD45^+^ cells colocalised with DAPI were counted at 200 × (in a 710 µm × 530 µm field) in each of the ischemic and contralateral hemispheres of 3 sections per mouse. For colocalization studies, data were presented as percentage of 3-NT^+^ cells of total F4/80^+^ cells. For analysis of GFAP expression, fluorescence intensity was analysed using ImageJ software (NIH, USA) in two 250 µm × 250 µm areas within the peri-infarct or corresponding contralateral regions, and normalised to the WT contralateral hemisphere. All appropriate secondary antibody controls were performed in the absence of corresponding primary antibodies to ensure that there was no nonspecific binding.

### Bacterial analysis in mouse lungs

Mice were killed by isoflurane inhalation overdose. Using sterile technique, lungs were removed and homogenised in 1 ml of sterile PBS. Ten µl of tissue homogenate was serially diluted 10-fold. Homogenates and their diluents were then pipetted onto brain heart infusion agar plates supplemented with 5% horse blood, and plates were incubated at 37 °C for 18 h after which bacterial colony forming units were counted.

### Quantitative real-time PCR

Some mice (n = 44) were killed by isoflurane inhalation overdose and perfused with sterile PBS (50 ml) via the left ventricle prior to decapitation and brain removal. Hemispheres were separated and immediately frozen in liquid nitrogen. Total RNA was extracted with TRIzol reagent (Thermofisher Scientific, USA) and RNeasy Mini Kit (Qiagen, Germany) followed by cDNA conversion with Quantitect Reverse Transcription kit (Qiagen). Quantitative RT-PCR was performed with TaqMan Gene expression primers (Applied Biosystems, USA) including *Il-1b*, *Tnf-a*, *Il-23a*, *Il-12a*, *Foxp3*, *Chil3*, *Il-10*, *Il-13*, *Tgfb*, and *IL-37* using Bio-Rad CFX96TM real-time PCR machine (Bio-Rad, USA). Data were expressed as fold change (2^−ΔΔCT^) relative to WT sham-operated mice, with the exception of IL-37 in stroke-operated mice, which was normalized to IL-37tg sham-operated mice.

### ELISA for IL-37 in human and mouse plasma

The level of circulating IL-37 in patients and mice was determined using a human IL-37 ELISA kit (Adipogen, Switzerland). Measurements were performed in duplicate and the results were averaged. Samples with obvious hemolysis, which was visually detected as a pink to red tinge, were not used for measurements. 100 µl of neat plasma was placed into wells coated with anti-human IL-37 antibody in duplicate. Plates were then sealed, incubated overnight at 4 °C and washed, followed by addition of Detection Antibody (Adipogen). Plates were washed again and incubated at 37 °C for 1 h. Horse radish peroxidase-conjugated anti-rabbit IgG was added, the plate was washed again and 3,3′,5,5′-tetramethylbenzidine substrate solution (Adipogen) was added to each well for 10 min in the dark to allow for colour development. Stop solution was then added and absorption was read at 450 nm by a CLARIOstar microplate reader (BMG Labtech, Germany). The standard curve was interpolated in a quadratic equation.

### Statistical analysis

Data are generally presented as mean ± standard error of the mean (S.E.M.). Statistical analysis was performed with GraphPad Prism 7.1 (GraphPad Software Inc, USA). Results were analyzed by two-tailed one-way analysis of variance (ANOVA) with Bonferroni post-hoc test, unpaired Student’s t-test or Welch’s t-test, as appropriate. In some cases, pairs of parameters collected from cohorts of patients or mice were assessed for correlation using Spearman’s rank correlation test. A ROUT test was conducted to exclude statistical outliers. The study was performed in a blinded manner wherever possible. As each animal was assigned to a code on the animal card for MCAO surgery, investigators were not strictly blinded to the genotype of animal during the surgery itself or during the clinical scoring (0–4) immediately prior to euthanasia. However, the post-stroke mobility was analysed using automated software, and therefore was unbiased. Furthermore, all subsequent analyses of tissues such as infarct volume, gene expression, immunohistochemistry and lung infection were performed in a blinded manner. *P* < 0.05 was considered statistically significant.

## Supplementary information

Suppl Figures

## Data Availability

The datasets generated during and/or analysed during the current study are available from the corresponding author on reasonable request.
