# Supplementary material for: IL-37 increases in patients after ischemic stroke and protects from inflammatory brain injury, motor impairment and lung infection in mice
Source: Sci Rep. 2019 May 6;9:6922. doi: 10.1038/s41598-019-43364-7 (PMC6502884; doi:10.1038/s41598-019-43364-7)
Supplement: Supplementary file 1 — Suppl Figures [file 41598_2019_43364_MOESM1_ESM.pdf]

IL-37 increases in patients after ischemic stroke and protects from inflammatory brain injury, motor impairment and lung infection in mice

Shenpeng R. Zhang[1,2], Marcel F. Nold[3,4], Sung-Chun Tang[5], Christine B. Bui[3,4], Claudia A. Nold[3,4], Thiruma V. Arumugam[6,7], Grant R. Drummond[1,2], Christopher G. Sobey[1,2 + *] and Hyun Ah Kim[1,2 +]

[1] Department of Physiology, Anatomy & Microbiology, School of Life Sciences, La Trobe University, Bundoora, Victoria, Australia;

[2] Cardiovascular Disease Program, Biomedicine Discovery Institute and Department of Pharmacology, Monash University, Clayton, Victoria, Australia;

[3] Ritchie Centre, Hudson Institute of Medical Research, Melbourne, Victoria 3168, Australia;

[4] Department of Paediatrics, Monash University, Melbourne, Victoria 3168, Australia;

[5] Department of Neurology, National Taiwan University Hospital, Taipei, 10002, Taiwan;

[6] Department of Physiology, Yong Loo Lin School of Medicine, National University of Singapore, Singapore;

[7] School of Pharmacy, Sungkyunkwan University, South Korea.

Running Head: Interleukin 37 in stroke

* Corresponding author

Email: c.sobey@latrobe.edu.au

[+] Contributed equally

Supplementary Figure S1: Regional cerebral blood flow (rCBF), infarct and edema volumes after stroke.

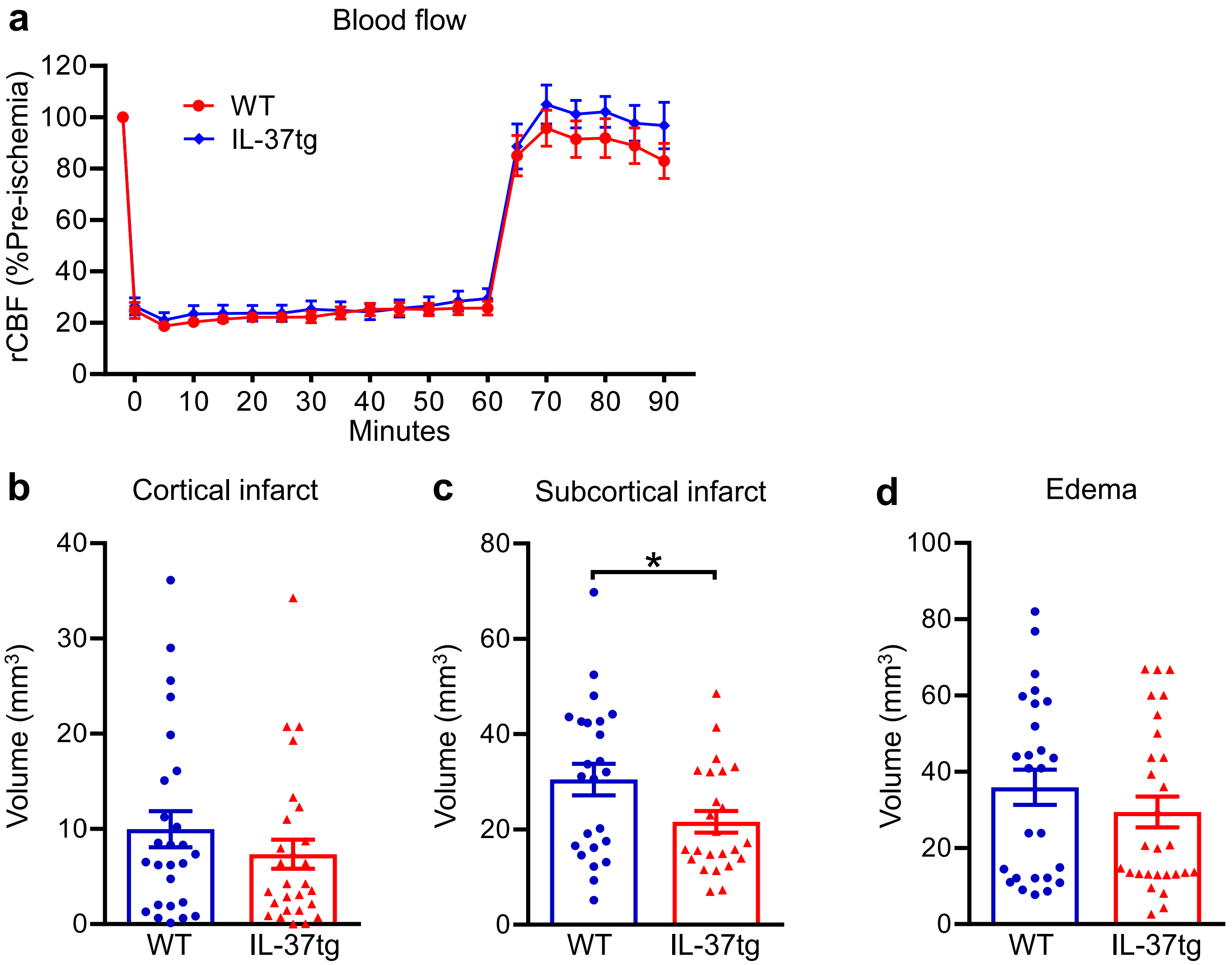

Supplementary Figure S1. Regional cerebral blood flow (rCBF), infarct and edema volumes after stroke. (a) rCBF during middle cerebral artery occlusion and reperfusion (n=20 in each of WT and IL-37tg mice). (b) Cortical, (c) subcortical and (d) hemispheric edema volumes in WT (n=26) and IL-37tg (n=28) mice at 24 h after stroke. Data are presented as mean ± S.E.M.; **P*<0.05, Welch's t-test for (b-d).

Supplementary Figure S2: Immunohistochemical quantification of immune cells and astrocytes in the ischemic hemisphere of WT mice after stroke.

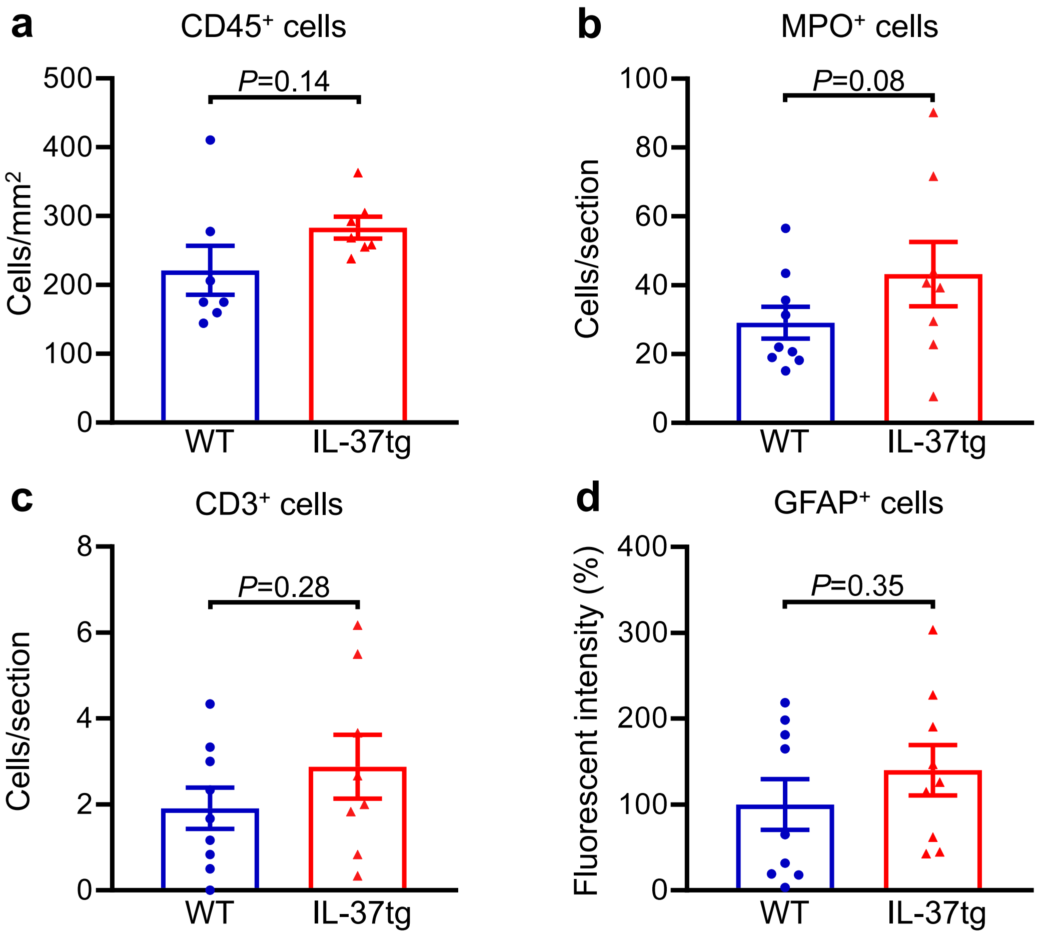

Supplementary Figure S2. Immunohistochemical quantification of immune cells and astrocytes in the ischemic hemisphere of WT (n=7-9) and IL-37tg (n=7-9) mice after stroke. Numbers of (a) $CD45^+$ leukocytes, (b) MPO^+ neutrophils, (c) $CD3^+$ T cells and (d) GFAP astrocytes at 24 h after stroke. Data are presented as mean ± S.E.M.; unpaired Student's t-test.
